# Consensus statement on the clinical application of extracorporeal shock wave therapy for diabetic foot ulcers (2025 Edition)

**DOI:** 10.1097/JS9.0000000000003489

**Published:** 2025-10-21

**Authors:** Yongtao Su, Xiaobing Fu, Yuesheng Huang

**Affiliations:** a Institute of Tissue Regeneration and Wound Repair, Shandong University of Traditional Chinese Medicine, Jinan, China; b Department of Wound Repair and Plastic Surgery, Shandong Provincial Hospital of Traditional Chinese Medicine, Jinan, China; c Research Center for Wound Repair and Tissue Regeneration, Medical Innovation Research Department, The PLA General Hospital, Beijing, China; d Institute of Wound Repair and Regeneration Medicine, Southern University of Science and Technology School of Medicine, Shenzhen, China

**Keywords:** diabetic foot ulcers, expert consensus, extracorporeal shock wave therapy

## Abstract

Diabetic foot ulcers (DFUs) are among the most severe late-stage complications in patients with diabetes. It is characterized by complex pathogenic factors, challenging treatments, and high rates of disability and mortality. Therefore, DFUs have become a major therapeutic challenge for patients with diabetes. In recent years, extracorporeal shock wave therapy (ESWT) has emerged as a novel modality for promoting the healing of both acute and chronic superficial wounds. An increasing number of studies have demonstrated significant clinical efficacy in the treatment of DFUs. Developed by experts from the Wound Repair Committee of the Chinese Medical Doctor Association through an extensive review of high-quality literature, this consensus systematically outlines the indications, contraindications, standardized procedures, key technical points, post-treatment patient management and assessment, and prevention and management of potential complications related to ESWT for DFUs. This consensus aims to provide clinical guidance and a practical reference for physicians in wound repair departments to apply ESWT in the management of diabetic foot ulcers.

**HIGHLIGHTS**

- This is the first national expert consensus on ESWT for diabetic foot ulcers in China.
- Recommendations were developed by 69 multidisciplinary experts using Delphi methodology.
- The consensus defines evidence-graded indications, contraindications, and clinical protocols.
- The document provides detailed parameters for ESWT use in various DFU classifications.
- This consensus bridges clinical evidence and practice for wound care professionals.

## Introduction

Diabetic foot ulcers (DFUs), one of the most severe complications of diabetes, stem from peripheral neuropathy and varying degrees of vascular disease in the lower extremities. It is characterized by foot infections, ulcerations, and deep tissue destruction. Clinically, DFUs manifest as infection, ulceration, degeneration, and necrosis of the foot tissues, and in severe cases, may lead to amputation.

Current treatment strategies for DFUs are primarily based on comprehensive internal medicine management supplemented by various approaches, depending on the patient’s clinical presentation. These include debridement and dressing changes, antimicrobial therapy, vascular reconstruction, hyperbaric oxygen therapy, negative pressure wound therapy, stem cell therapy, bioengineered skin substitutes, and surgical interventions. However, most DFU patients often present with multiple comorbidities, poor general health, and limited financial resources. This highlights the urgent need for a cost-effective, safe, and efficacious treatment modality. Extracorporeal shock wave therapy (ESWT) is a non-invasive, safe, and effective technique that has been widely applied in various medical fields^[1]^. In recent years, a growing body of literature has reported promising outcomes and broad prospects of ESWT in the management of DFUs^[2,3]^. Nevertheless, its widespread clinical adoption has been hindered by inconsistencies in application protocols, lack of standardized technical guidelines, and absence of unified treatment regimens.

To address these issues, the Wound Repair Committee of the Chinese Medical Doctor Association convened a panel of experts guided by evidence-based medicine and the Delphi method. Based on a comprehensive review of recent domestic and international literature on the use of ESWT in DFUs and drawing upon national clinical experience, the panel formulated this expert consensus. This document aims to provide a standardized reference for the clinical application of ESWT in the treatment of DFUs in China. In accordance with the TITAN 2025 Guidelines for transparent reporting of AI involvement in scientific work, this manuscript includes a completed TITAN checklist to ensure compliance and transparency^[4]^.

## Scope of application

This consensus provides guidelines for the use of ESWT in the diagnosis and treatment of DFUs, specifically in adult patients diagnosed with this condition. It addresses healthcare institutions and health management departments that provide services to this population. This consensus serves as a reference for healthcare professionals in wound repair, medical rehabilitation, endocrinology departments, and medical wellness institutions.

## Methods

### Consensus development group and their responsibilities

The consensus was pre-registered in both Chinese and English and was developed based on a prewritten protocol. The consensus working group consisted of 69 experts from multiple disciplines, including wound repair, burn and plastic surgery, endocrinology, and evidence-based medicine. The group was structured into the following subgroups: advisory committee, leadership team, expert panel, drafting team, literature review group, methodology team, evidence appraisal group and secretariat group. Experts from multidisciplinary fields were invited to form relevant groups through advisors and group leaders.

The literature review group was responsible for identifying high-quality studies related to DFUs and ESWT based on predefined clinical questions. Searches were conducted using both Chinese and English keywords such as “extracorporeal shock wave therapy,” “shock wave therapy,” “focused shock wave therapy,” “defocused shock wave therapy,” “radial shock wave therapy,” “wound healing,” “diabetic foot ulcer,” and “skin ulcer.” The databases searched included PubMed, Medline, Web of Science, Wanfang, and CNKI, with the search period covering the inception of each database to 1 December 2024. Relevant references were manually retrieved to supplement the electronic search. The scope of the search was limited to human disease-related studies, and eligible literature included systematic reviews, meta-analyses, randomized controlled trials (RCTs), cohort studies, case series, and expert consensus statements. The detailed literature search strategy is shown in Supplementary Digital Content Material 2, available at: http://links.lww.com/JS9/F363.

The methodological quality of the included RCTs was assessed using the Cochrane Risk of Bias tool; cohort and case-control studies were evaluated using the Newcastle-Ottawa Scale; and the Joanna Briggs Institute criteria were used to appraise case series and expert opinion-based evidence (see Table 1).

**Table 1:** Level of evidence

Level of evidence	Definition
A	The materials are derived from multicenter controlled clinical trials or meta-analyses.
B	The materials are derived from single-center controlled clinical trials or large-scale non-controlled studies.
C	Expert opinion and/or data from registered small-scale or retrospective studies.

Based on literature review and expert consultation, the drafting team formulated key clinical questions, which were then evaluated for their importance via an online survey. These questions were structured using the PICO format (P: Population/Patient, I: Intervention, C: Comparator, O: Outcome). A Delphi questionnaire was subsequently designed and distributed to experts. The collected data were analyzed and fed back to the expert panel to develop the initial draft of the consensus. This draft was then revised based on feedback and suggestions from the expert group. The methodology expert group was responsible for guiding the consensus plan writing and literature quality assessment, among other things. Supplementary Digital Content (Material 1, available at: http://links.lww.com/JS9/F362; Material 2, available at: http://links.lww.com/JS9/F363; Material 3, available at: http://links.lww.com/JS9/F364; Material 4, available at: http://links.lww.com/JS9/F365) provides the detailed Delphi questionnaire, search strategy, risk of bias and sensitivity analyses, and the TITAN Guideline Checklist used in this study.

### Formulation of consensus recommendations

The expert group formed its recommendations by assessing factors such as the quality of evidence, balance of benefits and harms, acceptability, and implementability, and based on the level of agreement to form the first draft of the recommendations for expert consensus. The recommendations were counted, summarized and given an appropriate level of recommendation based on the results of the deliberations after obtaining feedback from the experts in the first round. Specific suggestions from the first round of feedback were revised and incorporated after further discussion and individual consultation within the expert group. Additional modifications were made based on the expert input before the second round of evaluation. The results of the two rounds of the Delphi method (see Supplementary Digital Content Material 1, available at: http://links.lww.com/JS9/F362 – Delphi for details) expert questionnaire feedback and the results of the first consensus workshop discussions were summarized, and the recommendations were tallied and refined according to established rules. Subsequently, the Consensus Workshop was convened to review each entry individually in light of the latest clinical evidence to form the final recommendations. Consensus Criteria: Recommendations supported by more than 90% of experts were classified as strong recommendations. Those receiving 70%–90% support were classified as having moderate recommendations. Recommendations with 50% to 70% agreement were considered weak recommendations. Any recommendation with a degree of consistency of <50% were not be included.Detailed information on the recommendation grade rating can be found in Table 2.

**Table 2:** Recommendation grade

Recommendation grade	Definition
I	There is evidence and/or general agreement that a given treatment or intervention is beneficial, useful, and effective.
II	There is conflicting evidence and/or divergence of opinion regarding the usefulness and/or effectiveness of a given treatment or intervention.
IIa	Evidence and/or expert opinion favor the usefulness and/or effectiveness of the given treatment or intervention.
IIb	The evidence and/or expert opinion regarding the usefulness and/or effectiveness is insufficient.
III	There is evidence or consensus indicating that the given treatment or procedure is ineffective and may be harmful in some cases.

## Clinical issues and recommendations

### Clinical issues 1

Indications for the Use of ESWT in the Treatment of DFUs

#### Recommendation 1

ESWT can be routinely used in patients with first-onset Wagner grade 1 or 2 DFUs. (Level of Evidence: A; Strength of Recommendation: I)

#### Recommendation 2

ESWT can be routinely applied to patients with Wagner grade 3 DFUs following effective debridement and drainage, provided there is no exposed bone tissue, no osteomyelitis, and the infection is confined to the wound area or has improved to Wagner grade 1 or 2 after treatment. (Level of Evidence: C; Strength of Recommendation: IIb)

#### Recommendation 3

ESWT can be routinely applied to patients with traumatic ulcers without severe infection or gangrene after Wagner grade 4 or 5 post-amputation/toe amputation. (Level of Evidence: C; Strength of Recommendation: IIb)

#### Recommendation 4

For patients with DFUs accompanied by sinus tracts, ESWT should be administered only after adequate debridement and low-position drainage have been performed. (Level of Evidence: C; Strength of Recommendation: IIb)

##### Rationale

Studies have shown that in 2021, approximately 537 million people worldwide are living with diabetes, with a global prevalence of 6.3%^[5]^. DFUs refer to skin ulceration and/or deep tissue destruction in the foot or ankle region of diabetic patients caused by distal lower limb neuropathy and/or varying degrees of vascular disease. These lesions may or may not be accompanied by infection^[6]^. The incidence of DFUs is approximately 8.1%^[7]^, making it one of the most serious complications of diabetes.

Currently, the most widely used classification system for DFUs in both clinical practice and research is the Wagner classification^[8]^, originally proposed by Meggitt in 1976 and later popularized by Wagner (see Table 3). ESWT is an emerging modality in physical therapy. In 2017, the USA Food and Drug Administration approved the use of ESWT for the treatment of DFUs wounds for the first time^[9]^. A growing body of research suggests that ESWT can promote superficial ulcer healing by stimulating angiogenesis and granulation tissue formation^[10]^. Therefore, for patients with DFUs classified as Wagner grade 1 or 2, routine ESWT has been shown to provide favorable outcomes^[3,11–14]^. In contrast, for patients with Wagner grade 3 DFUs complicated by localized abscesses or osteomyelitis, the mechanical stress accompanying the shock waves may drive bacteria or toxins into deeper tissues or the bloodstream, potentially leading to systemic infections. Therefore, ESWT is not recommended in such cases ^[14]^. Similarly, in patients with Wagner grade 4 or 5 ulcers associated with localized gangrene, the cells in the gangrenous area are necrotic and lack regenerative capacity, and ESWT is unlikely to reverse tissue necrosis and is not recommended for these cases^[15]^. However, based on our clinical observations, ESWT may still be effectively applied in patients with Wagner grade 3 ulcers who have undergone adequate debridement and drainage, or in grade 4/5 patients who have undergone amputation or toe resection and have been down-staged to Wagner grade 1 or 2 ulcers. ESWT has shown promising results in these cases, and for DFUs cases with sinus tracts, thorough debridement and low-position drainage should precede the application of ESWT to prevent fluid accumulation.

**Table 3:** Wagner classification of diabetic foot ulcers

Classification	Clinical manifestations
Grade 0	Presence of risk factors for foot ulceration without current ulceration
Grade 1	Superficial foot ulcer without signs of infection, predominantly presenting as a neuropathic ulcer
Grade 2	Deeper ulcer often accompanied by soft tissue infection, without osteomyelitis or deep abscess formation
Grade 3	Deep ulcer with abscess formation or osteomyelitis
Grade 4	Localized gangrene (toes, heel, or forefoot), characterized by ischemic necrosis typically accompanied by neuropathy
Grade 5	Extensive gangrene of the entire foot

### Clinical issues 2

Contraindications for ESWT in the Treatment of DFUs

#### Recommendation 5

ESWT should be avoided in patients with new thrombosis (acute phase) in the lower extremity treatment area, acute phase fractures, malignant tumors, active wound bleeding, or exposed blood vessels following debridement. (Evidence level: C; Recommendation grade: IIa)

#### Recommendation 6

Shockwave therapy should be avoided in patients with significant coagulation disorders, implanted pacemakers, uncontrolled hypertension (systolic BP >180 mmHg or diastolic BP >110 mmHg), severe protein malnutrition (serum albumin <20 g/L), or severe anemia (hemoglobin <70 g/L). (Evidence level: C; Recommendation grade: IIa)

#### Recommendation 7

Patients with mental disorders, women who are pregnant or recently intended to become pregnant, and patients with systemic infections (e.g., sepsis) with DFUs should avoid shockwave therapy. (Evidence level: C; Recommendation grade: IIa)

##### Rationale

In an animal study^[16]^, researchers pointed out that the cavitation effect of shockwaves can cause thrombi or fat emboli to disintegrate and fragment, leading to vascular recanalization. When deep vein thrombosis is present locally, ESWT treatment may cause the thrombus to loosen, fragment, or even detach, ultimately resulting in pulmonary embolism or other diseases. A case reported in the literature^[17]^ indicated that shockwave therapy for ureteral stones led to spontaneous miscarriage, which may be related to the close proximity of the therapy site; however, ESWT should be avoided in pregnant women or those with recent pregnancy intentions (to avoid pregnancy without knowledge). In a study on shockwaves^[18]^, it was noted that there was an increased risk of hematoma when ESWT was administered to patients with coagulation disorders or those who were being treated with drugs such as warfarin. The study also reported three cases in which blood pressure increased during or after treatment, and one case involved a hypertensive crisis that led to syncope. Furthermore, if there is an infection site in the treatment area, shockwave therapy may stimulate the inflammatory focus, potentially exacerbating the condition. Therefore, treatment should be administered only after infection control. The article also suggests avoiding ESWT in patients with autonomic dysfunction or high levels of emotional anxiety. Shockwaves can cause local damage during treatment; therefore, so it be avoided if there is active exudation or exposed blood vessels in the treatment area, to prevent further injury. Several previous consensus guidelines^[1,19]^ recommend avoiding ESWT in patients with malignant tumors, severe protein malnutrition, or severe anemia, as these patients may not tolerate the treatment well.

### Clinical issues 3

Preoperative Assessment for the Use of ESWT in the Treatment of DFUs.

#### Recommendation 8

A comprehensive evaluation of the patient’s overall condition should be conducted prior to the initiation of ESWT for DFUs. This includes a thorough assessment of the patient’s general physical status, cognitive function, psychological expectations, social background, history of diabetes, history and progression of DFUs, and previous treatment outcomes. Such evaluations help determine the appropriateness of ESWT for individual patients. (Evidence Level: C; Recommendation Grade: IIa)

#### Recommendation 9

A careful assessment of the skin condition below the knee and the status of lower limb arterial pulses should be performed. At least one of the following vascular assessments should be completed: ankle-brachial index (ABI), transcutaneous oxygen pressure (TcPO₂), lower limb vascular ultrasound, computed tomography angiography (CTA), magnetic resonance angiography (MRA), or digital subtraction angiography (DSA). In addition, neuropathy evaluation should be conducted to help determine the prognosis and assess the effectiveness of ESWT. (Evidence Level: C; Recommendation Grade: IIa)

#### Recommendation 10

A comprehensive evaluation of the local DFUs should include the color, depth, anatomical location, exudate characteristics, granulation tissue growth, extent of infection, local temperature, and sensory function. These parameters serve as important indicators for assessing the therapeutic response to ESWT. (Evidence Level: C; Recommendation Grade: IIa)

##### Rationale

Prior to initiating treatment, both the systemic and local conditions of the patient should be thoroughly evaluated, incorporating both subjective and objective indicators. A patient’s confidence and adherence to treatment significantly impact therapeutic outcomes. Research indicates that 20%–40% of individuals with diabetes experience varying degrees of depression and anxiety^[20]^. These emotional disturbances are associated with an increased incidence and mortality of diabetic complications, which in turn slow the healing of DFUs. Maintaining optimal blood glucose levels is a fundamental prerequisite for promoting DFUs healing^[21]^. Poor glycemic control not only hinders ulcer recovery but also increases the risk of serious complications. Chronic hyperglycemia in DFUs patients often leads to neuropathy and peripheral vascular diseases. Therefore, treatment should not be limited to local ulcer care, but should also address neurological impairment and microcirculatory dysfunction^[22]^. In patients with significant lower extremity ischemia, vascular intervention can be performed first through the detection of relevant indices, which is of great significance to the prognosis of the patients^[23,24]^.

Additionally, peripheral neuropathy in diabetic foot requires careful management. Some researchers regard neuropathy as a primary factor that precipitates vascular lesions and impairs wound healing^[25]^. Positive outcomes have been reported with combined bone transport and neurolysis in the treatment of DFUs. Early ambulation under protective measures such as casting is also recommended post-neurolysis^[26,27]^. The severity, depth, and size of DFUs vary, as do the types of infecting bacteria^[28]^, treatment strategies should be individualized. Evaluating the local condition of an ulcer helps develop a personalized treatment plan. Continuous monitoring of local ulcer characteristics enables quantification of ESWT efficacy and provides objective data to adjust treatment parameters, including frequency, energy level, and duration.

### Clinical issues 4

Types of Extracorporeal Shock Waves Suitable for the Treatment of DFUs.

#### Recommendation 11

Radial extracorporeal shock wave therapy (r ESWT) has demonstrated superior efficacy over other methods for treating superficial soft tissue defects, making it particularly suitable for patients with DFUs. (Level of Evidence: B; Recommendation Grade: IIa)

#### Recommendation 12

Focused extracorporeal shock wave therapy (f ESWT) may be considered for the treatment of deep tissue injuries, such as wounds with sinus tracts complicated by osteomyelitis. Treatment parameters should be tailored based on wound characteristics and patient tolerance. (Level of Evidence: C; Recommendation Grade: IIb)

##### Rationale

Shock waves are discontinuous pressure waves that propagate through a medium. They occur when the source moves faster than the speed of sound in that medium, and are also referred to as shockwaves or blast waves^[29]^. Originally developed for extracorporeal lithotripsy in the treatment of urinary tract stones, ESWT has since expanded to other areas, including bone healing and pain management^[30]^.

Based on their generation mechanism, shock wave sources can be classified into electrohydraulic, piezoelectric, electromagnetic, and pneumatic ballistic types^[31]^. Depending on how the waves are transmitted, they are further categorized as focused shock waves or radial (divergent) shock waves, each with distinct physical characteristics^[32]^.

Focused shock waves and radial shock waves act on a small, concentrated area through mechanisms such as wave superposition and reflection. Focused shock waves are characterized by a high peak pressure (often exceeding 50 MPa, much higher than the ~15 MPa of radial waves), rapid rise time (5–10 ns), and a steep pressure gradient. They also penetrate deeply (up to >10 cm) and concentrate energy within a narrow focal zone (2–8 mm in diameter)^[29]^. Owing to their powerful penetrative capacity, focused shock waves are mainly used for conditions involving bone and deep soft tissues, such as nonunion fractures, calcific tendinitis of the rotator cuff, plantar fasciitis, and avascular necrosis of the femoral head^[33–35]^.

In contrast, radial shock waves are typically generated via mechanical impact, in which compressed air or electromagnetic force propels a metal projectile at high velocity to strike a transmitter (metal or plastic) at the tip of the device^[36]^. This kinetic energy is converted into shock waves that radiate spherically from the point of contact with the skin. Unlike focused waves, radial waves lack a distinct focal point and their energy dissipates quickly with distance. The highest energy is delivered near the skin surface and spreads over a broader area, primarily affecting superficial tissues^[37,38]^.

The key features of radial shock waves include a lower peak pressure (5–15 MPa, roughly one-third to one-fifth that of focused waves), slower pressure rise time (1–25 μs), gentler pressure gradients, weaker penetration (energy decreases sharply within 1–3 cm, with 90% concentrated within 2 cm of the surface), and broader energy dispersion (with coverage diameters up to 5 cm, making precise targeting difficult)^[39]^.

Owing to these properties, radial shock waves are more suitable as adjunct therapies in chronic wound care, particularly for promoting healing of DFUs and treating scar tissue. Therefore, patients with DFUs are better suited for treatment with radial (divergent) shock wave therapy.

### Clinical issues 5

Precautions Before Performing ESWT.

#### Recommendation 13

Patients should be provided with scientific knowledge of the principles and mechanisms of ESWT to enhance their confidence in the treatment process. Establish a philosophy of lifelong prevention and intervention is essential. Patients should be encouraged to return for follow-up visits, allowing for continuous monitoring of wound progression and early identification of potential complications. (Level of Evidence: C; Strength of Recommendation: IIa)

##### Rationale

Extracorporeal shockwave therapy (ESWT), a non-invasive modality utilizing high-energy acoustic waves, has shown promising clinical efficacy as an adjunctive treatment in diabetic foot ulcer (DFU) management^[9]^. Its therapeutic mechanism primarily involves the generation of mechanical stimulation and downstream biological responses at the treatment site, which together promote angiogenesis, enhance microcirculation, and accelerate wound healing, ultimately reducing the risk of ulcer recurrence.

Effective patient education is critical to optimizing ESWT outcomes in DFU. Patients should be informed of the underlying therapeutic mechanisms, including ESWT’s ability to stimulate cellular proliferation, promote the release of endothelial growth factors, and enhance collagen synthesis, all of which contribute to tissue repair^[40,41]^. Furthermore, its safety profile should be emphasized, as ESWT is associated with relatively few adverse effects and is generally well tolerated^[14]^.

Patient education should be contextualized within the broader framework of lifelong prevention and timely intervention, recognizing that diabetic foot represents a chronic, multifactorial complication closely linked to vascular insufficiency and peripheral neuropathy. Long-term, multidisciplinary management is often required. Patients should be encouraged to adopt sustained preventive strategies, particularly with regard to the tight regulation of glycemic control, blood pressure, and lipid levels. Concurrently, structured follow-up systems and regular wound surveillance are necessary. During and after ESWT, patients should be advised to monitor for any changes in wound size, exudate volume, or signs of infection, and to seek medical attention promptly for individualized adjustment of therapeutic parameters if needed^[42]^.

In addition to procedural counseling, lifestyle modification remains a cornerstone of DFU management. Nutritionally, patients should adhere to diabetes-specific dietary guidelines, focusing on controlled caloric intake and balanced macronutrient composition to mitigate the deleterious effects of hyperglycemia on wound healing. Environmental and self-care guidance is also crucial: patients should maintain a warm and dry living environment to avoid impairments in circulation or increased infection risk, and exercise caution during foot hygiene to prevent thermal injury. Smoking cessation must be strongly advocated, as nicotine-induced vasoconstriction impairs tissue oxygenation and delays healing. Physical activity should be appropriately calibrated – while excessive weight-bearing on the affected limb must be avoided, moderate, low-impact activities such as balance exercises or slow walking may enhance local perfusion and systemic metabolic health^[43]^.

Proper footwear and pressure offloading are essential for minimizing mechanical stress and preventing ulcer exacerbation. Patients should be instructed to wear custom offloading shoes or insoles and to perform daily inspections of both foot surfaces and footwear interiors for early signs of trauma, such as blisters, fissures, or erythema, warranting immediate attention^[44]^.

Collectively, these educational components – integrated with ESWT – constitute a critical element of comprehensive DFU management. Through close physician–patient collaboration, interdisciplinary care, and sustained health education, the therapeutic benefits of ESWT can be fully leveraged to promote wound healing, reduce the risk of recurrence and amputation, and significantly enhance patient quality of life^[45]^.

#### Recommendation 14

Provide patients with education on healthy lifestyle modifications, including adherence to a diabetic-friendly diet, maintaining a warm and comfortable living environment, avoiding smoking and excessive weight-bearing on the affected limb, wearing offloading footwear, and appropriately increasing physical activity levels. (Level of Evidence: C; Strength of Recommendation: IIa)

##### Rationale

Wound irrigation plays a critical therapeutic role in the treatment of DFUs. Techniques such as fluid lavage, medicated irrigation, and closed drainage can effectively remove necrotic tissue, foreign bodies, and inflammatory exudates from the ulcer site. This helps reduce the local microbial load, cleanse the wound, and create an environment conducive to healing^[46]^.

Debridement by autolytic or “conservative sharp” methods allows for the gradual removal of devitalized tissue without causing pain or bleeding. This approach minimizes additional trauma and eliminates key barriers to healing^[47]^.

Medical ESWT typically requires a fluid medium, such as water, or coupling agents to facilitate wave transmission into biological tissues^[48]^. These media help minimize energy loss during propagation, ensure that shock waves reach the targeted treatment area more effectively, and enhance the therapeutic effect.

It is recommended to apply a coupling gel between the shock wave probe and the skin to eliminate air bubbles, which could otherwise interfere with energy transmission and reduce therapeutic efficacy^[49,50]^.

In the treatment of burn wounds, where the skin barrier is often compromised, improper sterilization of the probe or repeated use of non-sterile coupling gel may increase the risk of secondary infection^[51]^. Therefore, it is advisable to protect the probe head using a sterile membrane before treatment. Moreover, it is also recommended that the coupling agent should use small sterile packages to avoid cross-infection caused by repeated use.

#### Recommendation 15

Before initiating ESWT, the patient’s skin condition and overall physical status were assessed. Ensure that the patient is fully informed about the potential risks associated with the procedure and obtain written informed consent. (Evidence Level: C; Recommendation Grade: IIa)

##### Rationale

A thorough patient assessment is essential prior to the initiation of ESWT. Careful examination and photographic documentation of the treatment site and surrounding skin should be performed. Due to the disruption of the skin’s physical barrier in conditions such as acute eczema, contact dermatitis, or radiation dermatitis, these inflammatory dermatoses are particularly susceptible to secondary infection^[52]^, and shockwave stimulation may exacerbate symptoms. In patients with purpura, telangiectasia, varicose veins, or coagulopathies (e.g., hemophilia), ESWT may increase the risk of bleeding or ecchymosis.

Patients with allergic predispositions or heightened pain sensitivity may experience discomfort during treatment and may require either avoidance of ESWT or modification to a gentler protocol. Clinicians must carefully evaluate the characteristics of the wound and surrounding tissues to select appropriate energy levels, thereby minimizing the risk of iatrogenic injury.

Given the complications reported in clinical practice – such as mild pain, numbness, ecchymosis, and irritant contact dermatitis^[53]^ – patients should be adequately informed of potential adverse effects and comprehensive informed consent should be obtained prior to treatment.

### Clinical question 6

Key Considerations During the Application of ESWT in Patients with DFUs: Treatment Areas, Frequency, and Intensity

#### Recommendation 16

If localized swelling, pain, or petechiae/ecchymosis occurs during treatment, the procedure should be stopped immediately. Appropriate topical medications and protective dressings should be applied. Mild redness, pain, or swelling are generally self-limiting and tend to resolve shortly after treatment. However, if the symptoms persist or worsen, further evaluation is warranted. (Evidence Level: C; Recommendation Grade: IIa)

##### Rationale

High-energy ESWT may cause damage to normal tissue cells, potentially leading to inflammation and necrosis^[31]^. Individual tolerance to treatment varies significantly. Some patients may develop localized swelling, pain, or bruising even from minor trauma during daily activities, for example, individuals with vitamin K deficiency^[54]^. Reports have documented adverse reactions such as local ecchymosis and hematoma in elderly patients undergoing ESWT for primary sarcopenia; however, these symptoms typically resolve after appropriate management^[55]^.

Transient pain following ESWT may be associated with the pro-inflammatory effects of therapy. Therefore, the careful administration is crucial. If a patient experiences discomfort during treatment, the procedure should be suspended, and appropriate symptomatic management should be initiated. If the patient improves, treatment may be resumed using safer parameters or alternative modalities. If symptoms persist despite treatment, a specialist evaluation is recommended to rule out underlying systemic conditions and facilitate appropriate adjustments to the therapeutic plan.

#### Recommendation 17

During shock wave therapy, particular attention should be paid to pressure adjustment, prioritizing patient tolerance. The treatment frequency should be modified according to the characteristics of the wound; lower frequencies are advised for deeper ulcers, while higher frequencies may be more appropriate for superficial lesions. Suggested treatment parameters include a pressure range of 1.0–2.5 bar and a frequency range of 8–13 Hz. (Level of Evidence: A; Recommendation Grade: IIa)

##### Rationale

According to current studies, the therapeutic effects of ESWT are energy-dependent. Low- to medium-energy shock waves have been shown to promote the release of nitric oxide and stimulate the polarization of macrophages from the pro-inflammatory M1 phenotype to the anti-inflammatory M2 phenotype, thereby exerting anti-inflammatory and analgesic effects. However, high-energy shock waves may cause significant tissue damage during treatment, including local inflammation, necrosis, hemorrhage, and potential nerve injury^[56]^.

In vitro experiments have demonstrated that a dose of 0.10 mJ/mm^2^ applied 500 times significantly promotes the proliferation of HaCat cells derived from chronic diabetic wounds^[57]^. Moretti *et al* found that at an energy flux density of 0.03 mJ/mm^2^, patients with DFUs in the treatment group showed superior wound healing time, closure rate, and epithelial regeneration index compared to controls^[58]^. Similarly, a clinical study by Omar *et al*^[12]^ reported that ESWT at an energy flux density of 0.11 mJ/cm^2^ significantly reduced wound size and the median time to ulcer healing, without adverse effects.

It is important to note that radial (dispersive) and focused shock waves use different units to express energy flux density – bar for radial waves and mJ/mm^2^ for focused waves. Some studies suggest that a radial wave at 2.0 bar is approximately equivalent to a focused wave at 0.09 mJ/mm^2^^[59]^. Given that DFUs are typically superficial, and that higher frequencies correspond to shallower zones of action within an appropriate pressure range, we recommend adjusting treatment parameters within the following energy ranges: Radial shock waves: 1.0–2.5 bar Focused shock waves: 0.03–0.18 mJ/mm^2^ Pressure should be tailored to the patient’s tolerance and absence of significant discomfort. Frequency should be selected based on pre-treatment wound evaluation: higher frequencies for more superficial wounds, with a general adjustment range of 8–13 Hz.

#### Recommendation 18

It is recommended that the treatment area include the wound site as well as a 2–3 cm margin surrounding the lesion. Alternatively, the treatment range can be guided by imaging modalities, such as ultrasound, CT, or MRI, to ensure accurate targeting. However, care should be taken to avoid applying shock waves directly over the major nerves and blood vessels. (Level of Evidence: B; Strength of Recommendation: IIa)

##### Rationale

Existing consensus^[19]^ has emphasized that accurate localization is a prerequisite for achieving optimal outcomes with ESWT, particularly in the treatment of musculoskeletal disorders. Common localization methods include surface anatomical landmarks combined with tender points, as well as imaging techniques such as X-ray, ultrasound, and magnetic resonance imaging (MRI). The treatment sites should avoid major blood vessels and the critical nerve trunks, and internal fixation devices should not obstruct the shock wave path.

Peripheral neuropathy, peripheral arterial disease, and local infections contribute to the development of DFUs. Lower limb vascular ultrasound can help assess the status of local vessels, whereas CT and MRI are useful for determining the extent of infection and for evaluating the presence of osteomyelitis. When used together, these imaging tools support the development of a comprehensive treatment strategy and help delineate the appropriate treatment area. In current DFUs treatment protocols, Jeppesen *et al*^[11]^ have defined the treatment region as the ulcer and a 1 cm margin surrounding it.

Previous studies have demonstrated that low-energy shock waves can improve the perineural microenvironment, enhance nerve conduction velocity, and aid in the recovery of motor function in several ways^[60,61]^. Additionally, they may suppress the release of pain-related neurotransmitters at nerve endings, thus alleviating pain symptoms^[62]^. However, some patients have reported transient numbness and other sensory disturbances after treatment^[63]^. Research also suggests that mechanical stimulation from shock waves may trigger localized inflammatory responses in skeletal muscle injury sites during the early phase^[64]^. While the cavitation effect of ESWT can enhance microcirculation, promote local metabolism, and reduce inflammation, its direct impact on blood vessels may cause new vascular injuries.

Therefore, this consensus recommends avoiding direct application of shock waves over major nerves and blood vessels to minimize patient discomfort and prevent potential complications.

#### Recommendation 19

It is recommended that the total number of shock wave impulses per treatment session be calculated as follows: 500 impulses plus an additional 100 impulses for each square centimeter of the treatment area. The number of impulses applied to a single treatment point should not exceed 100, and the treatment should proceed sequentially across the affected area. The recommended treatment frequency is 2 to 3 sessions per week, with a course of treatment lasting 4 weeks. The duration and number of treatment courses should be individualized based on the patient’s recovery progress. For patients with milder conditions or those whose DFUs have improved but not fully healed after other interventions, outpatient ESWT sessions are appropriate and effective. (Evidence Level: B; Recommendation Grade: IIa)

##### Rationale

Animal studies have demonstrated that for chronic skeletal muscle injuries in rats, ESWT achieves optimal reparative effects at an energy flux density of 0.14 mJ/mm^2^, with a frequency of 10 Hz and 500 impulses per session^[64]^. In a randomized controlled clinical trial, Omar *et al*^[12]^ found that administering ESWT twice weekly for 4 weeks, with 100 impulses per cm^2^, significantly reduced wound size and average healing time in patients with DFUs. Similarly, Wang *et al*^[65]^ reported favorable outcomes when DFUs patients received ESWT once every 2 weeks for three sessions, using a dosage of 300 + 100 impulses per cm^2^.

For chronic leg ulcers, other studies^[66]^ have suggested tailoring the number of impulses to the ulcer surface area. Under a regimen of 300 + 100 impulses per cm^2^ every three days, most ulcers healed effectively, with notable pain relief. ESWT is known to exert cumulative and time-dependent therapeutic effects^[67]^. For example, Ge *et al*^[68]^ demonstrated significant improvements in shoulder soft tissue injuries after 4 weeks of ESWT.

Furthermore, Kamelger *et al*^[69]^ investigated the dose-dependent response of ESWT by varying pulse counts (200, 500, 1500, 2500, 5000, and 10 000) at a fixed energy density of 0.1 mJ/mm^2^. Their findings showed the most pronounced therapeutic benefit at 500 impulses, with no significant gains at 1500 impulses and signs of tissue necrosis appearing at 5000 impulses.

The fundamental mechanism of ESWT lies in its ability to induce localized microtrauma, thereby triggering an inflammatory response and activating natural repair mechanisms. However, repeated shockwave application to the same treatment point or area can cause excessive tissue damage, potentially overwhelming the regenerative capacity of the body and resulting in further injury.

Based on a synthesis of domestic and international studies and clinical experience, we recommend a treatment protocol of 500 impulses plus 100 impulses per cm^2^ of wound area per session. No more than 100 impulses should be applied at any point. Sessions should be scheduled 2–3 times per week, with one treatment cycle lasting 4 weeks. Given the variability in disease severity and patient compliance, ESWT should be tailored to each individual, ensuring comprehensive DFUs care while allowing for flexible outpatient treatment options.

### Clinical question 7:

Post-Procedure Considerations After ESWT

#### Recommendation 20

After shock wave therapy, the coupling agent was removed from the wound, disinfection was performed again, and appropriate dressings were selected based on the local condition of the wound. Consider adding antibiotic ointment, growth factors, or other relevant treatments as needed. In the early stages of shock wave therapy, wound exudates may increase; therefore, it is recommended to use absorbent dressings to cover the wound. This helps avoid excessive moisture while reducing the dressing change frequency. (Evidence Level: C; Recommendation Level: IIa)

##### Rationale

In a clinical study of pressure injury ulcers^[70]^, researchers pointed out that some patients experienced an increase in ulcer size and exudate during the early stages of ESWT treatment. The reason was thought to be the failure to assess the extent of ischemic tissue, focusing only on the size of the skin damage. While the ulcer area increased and exudate increased, the wound healing speed accelerated in the later stages. This suggests that ESWT can also serve as a non-surgical debridement method. The theory of moist wound healing states that when a wound is in a moist environment, tissue dehydration and cell death are reduced, blood vessel regeneration speed increases, and growth factors accelerate wound repair, thereby promoting epithelial cells migration^[71]^. However, excessively moist wounds contain high levels of reactive oxygen species and matrix metalloproteinases, which can hinder cell recruitment, extracellular matrix reconstruction, and angiogenesis^[72]^. Because the coupling agent is a foreign substance in the wound healing process, it should be cleaned after treatment. Based on the condition of the wound bed, appropriate dressings, antibiotic ointments and growth factors should be selected. Due to increased exudate in the early stages, it is important to promptly replace soaked dressings and use highly absorbent dressings (such as foam dressings, alginate dressings, or silver ion dressings) to cover the wound, preventing maceration that could affect healing.

#### Recommendation 21

Throughout the course of ESWT, it is essential to maintain comprehensive and standardized foundational care. This includes routine glycemic control, nutritional support, infection management, prevention and treatment of neuropathy, appropriate foot weight bearing, and adjunctive use of vasodilators in patients with lower extremity stenosis. (Level of Evidence: A; Recommendation Grade: I)

##### Rationale

The pathogenesis of DFUs is multifaceted and involves impaired glucose metabolism and peripheral neurovascular complications. Factors such as hyperglycemia, chronic inflammation, local hypoxia, and neuropathy can alter cellular behavior and cytokine expression at the ulcer site, collectively contributing to delayed wound healing^[73]^. Currently, single-modality treatments often yield limited efficacy. Therefore, the clinical management of DFUs typically involves a multidisciplinary approach that integrates systemic medical therapy with localized interventions. Treatment should be tailored to the patient’s condition and may include standard glycemic control, nutritional support, infection management, neuropathy prevention and treatment, appropriate offloading of the foot, and the use of vasodilators in patients with lower extremity vascular stenosis. This comprehensive strategy is critical for reducing both amputation rates and mortality.

#### Recommendation 22

If there is no noticeable improvement in the wound or overall healing after 2–3 sessions of ESWT, a comprehensive reassessment of both the wound and the patient’s general condition should be conducted. Based on these findings, the treatment plan should be adjusted in a timely manner to prevent further deterioration or progression of the condition. (Level of evidence: C; Strength of recommendation: IIa)

##### Rationale

Shock wave therapy exhibits a cumulative dose-dependent effect^[74]^, and its therapeutic benefits may not be immediately apparent. Over time and with repeated sessions, the effects typically become more pronounced. For patients who do not respond well to the initial treatment, a follow-up course of therapy can be scheduled, during which treatment parameters should be appropriately adjusted. It is important to document patients’ subjective experiences and changes in their wound bed conditions. If no improvement is observed upon re-evaluation, alternative treatment strategies should be promptly considered to prevent further deterioration of the condition.

#### Recommendation 23

After ESWT, patients should avoid prolonged walking, intense physical activity, or heat-based physiotherapy. These actions may lead to local hyperemia or bleeding and should be prevented. (Level of Evidence: C; Recommendation Grade: IIa)

##### Rationale

When shock waves reach a certain intensity, their cavitation and thermal effects enhance microcirculation and inhibit depolarization, thereby producing anti-inflammatory and analgesic effects. However, from a broader perspective, they may also induce local hyperemia. Therefore, patients should avoid vigorous physical activity or physiotherapy immediately after treatment to prevent excessive local blood flow or potential bleeding^[75]^.

### Clinical question 8

The Combined Use of ESWT with Other Treatment Modalities in the Management of DFUs

#### Recommendation 24

Adjunctive therapies may promote wound healing during the course of treatment. These include agents that improve microcirculation (e.g., alprostadil and beraprost sodium), antiplatelet medications (such as aspirin and clopidogrel), growth factors, and topical antibiotic ointments. (Level of Evidence: B; Strength of Recommendation: IIa)

#### Recommendation 25

Phototherapy using red and blue light may be employed as an adjunctive treatment to reduce the bacterial load at the wound site, control infection, and promote healing.(Level of Evidence: B; Strength of Recommendation: IIa)

#### Recommendation 26

Administering ESWT 24 hours after perilesional injection of platelet-rich plasma (PRP) may enhance PRP diffusion and accelerate wound healing.(Level of Evidence: B; Strength of Recommendation: IIa)

#### Recommendation 27

Combined use of WIRA and diabetic foot electrotherapy devices can more effectively reduce pain, improve blood circulation, and suppress inflammation. However, immediate application should be avoided, with a treatment interval of more than 24 hours.(Level of Evidence: B; Strength of Recommendation: IIa)

##### Rationale

When treating DFUs, various combination therapies have been recommended to promote wound healing. First, during the treatment process, oral or topical medications can be used, such as drugs to improve microcirculation (e.g., prostaglandin E1, beraprost sodium), antiplatelet drugs (such as aspirin, clopidogrel), and topical growth factors and antibiotic ointments to enhance wound healing^[12,76,77]^. Additionally, applying ESWT 24 hours after the injection of PRP around the skin lesions can promote the diffusion of PRP and wound healing^[78]^. PRP contains a high concentration of platelets and various growth factors that promote tissue repair and regeneration. In contrast, ESWT stimulates angiogenesis and tissue remodeling through mechanical stimulation. The combined use of these therapies can work synergistically to accelerate the wound healing process.

The combined application of ESWT and physical therapy also shows promising results. Red and blue light therapy can reduce bacterial load on the wound, control infection, and promote wound healing^[79]^. The WIRA, which operates in the 580–1200 nm wavelength range, penetrates up to 7 cm below the skin surface, providing deep tissue treatment. It is important to note that while therapies such as WIRA and diabetic foot electrotherapy devices can reduce pain, improve blood circulation, and suppress inflammation, they should not be used immediately in conjunction with ESWT. The treatment interval should be greater than 24 hours^[80]^.

In summary, the combined use of ESWT and other therapies offers multiple effective options for the treatment of DFUs. In clinical practice, these treatments should be carefully selected and tailored based on the patient’s specific condition and the characteristics of the wound to achieve the best therapeutic outcomes.

### Clinical question 9

Management of Infected Wounds

#### Recommendation 28

For infected wounds, routine sampling for bacterial culture and drug sensitivity testing should be performed before shockwave therapy. Appropriate antibiotics should be selected based on the severity and location of the infection. (Evidence Level: C; Recommendation Level: IIb)

#### Recommendation 29

For infected wounds, thorough debridement should be performed before shockwave therapy to remove necrotic tissue and exudates. The wound should then be covered with a sterile film, and coupling gel applied. Local debridement and drainage are fundamental to treatment. (Evidence Level: B; Recommendation Level: IIb)

#### Recommendation 30

For infected wounds, appropriate antibiotic ointments, such as mupirocin ointment, fusidic acid cream, or combined polymyxin ointment, can be selected based on epidemiological characteristics or drug sensitivity. These ointments may be applied directly to the wound instead of coupling gel, followed by coverage with a sterile film. At the same time, ensure that the shockwave therapy head is protected with a sterile film to prevent any air bubbles between the therapy head and the wound during treatment. (Evidence Level: C; Recommendation Level: IIb)

##### Rationale

The pathogens responsible for infected wounds are complex, with significant variations in antibiotic resistance. Multiple studies have shown that failure to use empirical antibiotics can lead to treatment failure or the emergence of antibiotic-resistant strains. A systematic review indicated that combining routine bacterial culture with drug sensitivity testing significantly improves the healing rate of infected wounds and reduces the risk of antibiotic misuse^[15]^. Moreover, for infections in specific areas such as diabetic foot or burn wounds, selecting antibiotics based on drug sensitivity results can shorten healing time^[81]^. Thorough debridement is a core measure in controlling infection. Research shows that debridement reduces bacterial load on the wound and promotes granulation tissue formation^[82]^. Sterile film coverage can isolate the wound from external contamination, maintaining a moist wound environment. A randomized controlled trial (RCT) showed that after debridement, using sterile film with coupling gel reduced the wound infection rate by 32% compared to the non-covered group^[83]^. Mupirocin, Fusidic acid, and combined polymyxin ointments exhibit potent bactericidal activity against common wound pathogens such as *Staphylococcus aureus* and *Pseudomonas aeruginosa*. A multicenter study demonstrated that locally applied mupirocin ointment increased the bacterial clearance rate in MRSA-infected wounds to 85%^[84]^. The combination of polymyxin ointment and shockwave therapy enhances the antimicrobial effect, potentially owing to the promotion of drug penetration by shockwaves^[85]^. The sterile film covering the therapy head helps avoid cross-contamination, ensuring treatment safety. Before shockwave therapy, it is crucial to ensure that no air bubbles are present in the wound to prevent energy attenuation. In vitro experiments have confirmed that energy loss from shockwaves can reach 30%–50% in the presence of bubbles, which impacts the therapeutic effect^[39]^. Therefore, strict adherence to aseptic techniques and standardized procedures is essential to optimize treatment outcomes.

## Conclusions

Currently, limited clinical evidence and a lack of rigorously designed studies leave key areas such as optimal shockwave therapy parameters, treatment duration, and the most suitable wound types for shockwave treatment without unified standards. Although ESWT has made progress in treating DFUs, the biological mechanisms underlying the effects of shockwaves on wound healing are not fully understood^[10,86]^. Current research suggests that the impact of shockwave therapy on wound healing is a complex, well-coordinated cascade process. However, during the development of this consensus, it became evident that evidence for ESWT in treating DFUs is still relatively scarce, necessitating multicenter, large-scale clinical studies. These studies should clarify the biological mechanisms by which ESWT achieves clinical efficacy at the tissue, cellular, and molecular levels. Expanding the clinical application of shockwave therapy, along with the standardization of ESWT training and certification for treatment institutions, should be prioritized. Professional committees related to shockwave medicine have already been established both domestically and internationally. It is believed that in the near future, as ESWT protocols continue to improve, they will serve as an effective adjunctive treatment, offering significant benefits to a broader population of diabetic foot patients.

## Data Availability

No datasets were generated or analyzed for this work. Data sharing is not applicable to this article.

## Research registration unique identifying number (UIN)

International Practice Guidelines Registry Platform: PREPARE-2025CN072.

## Evidence evaluation

Yuesheng Huang from the Institute of Wound Repair and Regenerative Medicine, School of Medicine, Southern University of Science and Technology; Zhixin Zhang and Guoyan Ren from the Institute of Tissue Regeneration and Wound Repair, Shandong University of Traditional Chinese Medicine.

## Secretary group

Jian Wang, Wei Zhang, Xiao Liu, Xuerong Zhang, and Shiyue Zhang from the Department of Wound Repair and Plastic Surgery, Affiliated Hospital of Shandong University of Traditional Chinese Medicine.

## Literature review group

Haoran Yin,Yifei Sun,Yuwei Chen from the Department of Wound Repair and Plastic Surgery, Affiliated Hospital of Shandong University of Traditional Chinese Medicine.

## Consensus consultants

The PLA General Hospital, Xiaobing Fu.

## Team leader

Southern University of Science and Technology Hospital, Yuesheng Huang.

## Consensus committee group

Hailin Xu from the Department of Trauma and Orthopedics, Peking University People’s Hospital; Hui Chen from Beijing Jishuitan Hospital; Junli Zhou from the Department of Burn, Plastic, and Wound Repair, Dongguan People’s Hospital; Yunchuan Pan from the Department of Burns and Wound Repair, Hainan Provincial People’s Hospital; Binghui Li from the Department of Wound Repair, Liyuan Hospital, Tongji Medical College, Huazhong University of Science and Technology; Hongwei Liu from the Department of Plastic Surgery, The First Affiliated Hospital of Jinan University; Biao Cheng from the Department of Plastic Surgery, General Hospital of Southern Theater Command of the PLA; Yufeng Jiang and Rungong Yang from the Department of Wound Repair, First Medical Center, Chinese PLA General Hospital; Jing Li from the Department of Plastic Surgery, Tangdu Hospital, Air Force Medical University; Guangping Liang from the Institute of Burn Research, First Affiliated Hospital, Army Medical University (Third Military Medical University); Jiaping Zhang from the Department of Plastic Surgery, First Affiliated Hospital, Army Medical University (Third Military Medical University); Jianhua Zhan from the Department of Burns and Wound Repair, The First Affiliated Hospital of Nanchang University; Yi Zhang from the Department of Burns and Wound Repair, Affiliated Hospital of Nantong University; Yibing Wang from the Department of Burns and Wound Repair Surgery, First Affiliated Hospital of Shandong First Medical University (Shandong Provincial Qianfoshan Hospital); Yongtao Su from the Institute of Tissue Regeneration and Wound Repair, Shandong University of Traditional Chinese Medicine; Yixin Zhang from the Department of Reconstructive Surgery, Ninth People’s Hospital, Shanghai Jiao Tong University School of Medicine; Yiwen Niu from the Wound Repair Center, Ruijin Hospital, Shanghai Jiao Tong University School of Medicine; Shuhua Liu from the Department of Burns, Wuhan Tongren Hospital (Wuhan Third Hospital), Wuhan University; Weiguo Xie from the Department of Burns, Wuhan Third Hospital; Xiaolong Liu from the Department of Burns and Plastic Surgery, People’s Hospital of Xinjiang Uygur Autonomous Region; Guoxian Chen from the Department of Burns and Wound Repair, The Second Affiliated Hospital, Zhejiang University School of Medicine; Pihong Zhang from the Department of Burns and Plastic Surgery, Xiangya Hospital, Central South University.

## Methodology expert group

Yueran Su from the School of Information Engineering, Haidu College, Qingdao Agricultural University; Xianwei Zhu, Zhixin Zhang, and Ying Sui from the Institute of Tissue Regeneration and Wound Repair, Shandong University of Traditional Chinese Medicine; Yuexin Su from the Academy of Mathematics and Systems Science, Chinese Academy of Sciences.

## Writing group

Haoran Yin, Yongtao Su, and Yifei Sun from the Department of Wound Repair and Plastic Surgery, Affiliated Hospital of Shandong University of Traditional Chinese Medicine; Yuesheng Huang from the School of Medicine, Southern University of Science and Technology.

## Clinical problem solicitation expert group

Chunlei Wang and Bo Zhang from the Department of Wound Repair, Burn, and Skin Surgery at Peking University Medical Luzu Hospital; Shouyu Wang from the First Affiliated Hospital of Dalian Medical University; Xiaowen Peng from Foshan First People’s Hospital; Jiayu Lin from the Second Affiliated Hospital of Fujian Medical University; Lijun Zou from Liyuan Hospital, Tongji Medical College, Huazhong University of Science and Technology; Haoyang Duan from the First Hospital of Jilin University; Chengzhi Li from the First Affiliated Hospital of Jinan University; Fuyong Wang from Kaifeng Central Hospital; Xin Wang from Ningbo Sixth Hospital; Lei Zhu from Qilu Hospital of Shandong University; Zhongzheng Guan and Wei Jiao from the Department of Wound Repair and Plastic Surgery, Shandong Provincial Hospital of Traditional Chinese Medicine; Guangtao Huang from Shenzhen Second People’s Hospital; Yueming Liu from Shenzhen Longhua District Central Hospital; Xiaoting Han from the General Hospital of Taiyuan Iron and Steel (Group) Co., Ltd.; Dalun Lv from the First Affiliated Hospital of Wannan Medical College; Ke Tao from the First Affiliated Hospital of Wenzhou Medical University; Man Luo from Wuhan Third Hospital; Zhiwei Ren from the First Affiliated Hospital of Xi’an Jiaotong University; Tao Song from Xi’an Red Cross Hospital; Li Li from the Fourth Affiliated Hospital of Xinjiang Medical University; Xingang Wang from the Second Affiliated Hospital of Zhejiang University School of Medicine; Li Zhang from the Second Medical Center of the Chinese People’s Liberation Army General Hospital.

## References

[1] Zhixiang C Xiaochong F Zhiying F. Chinese expert consensus on the clinical application of extracorporeal shock wave therapy (2023 Edition). Chin J Pain Med 2023;19:220–35.

[2] Galiano R Snyder R Mayer P Rogers LC Alvarez O. Focused shockwave therapy in diabetic foot ulcers: secondary endpoints of two multicentre randomised controlled trials. J Wound Care 2019;28:383–95. 31166864 10.12968/jowc.2019.28.6.383

[3] Snyder R Galiano R Mayer P Rogers LC Alvarez O. Diabetic foot ulcer treatment with focused shockwave therapy: two multicentre, prospective, controlled, double-blinded, randomised phase III clinical trials. J Wound Care 2018;27:822–36. 30557108 10.12968/jowc.2018.27.12.822

[4] Riaz AA Ginimol M Rasha R. Transparency in the reporting of Artificial Intelligence – the TITAN Guideline. Prem J Sci 2025;10:100082.

[5] Zhang P Lu J Jing Y Tang S Zhu D Bi Y. Global epidemiology of diabetic foot ulceration: a systematic review and meta-analysis (†). Ann Med 2017;49:106–16. 27585063 10.1080/07853890.2016.1231932

[6] Margolis DJ Malay DS Hoffstad OJ. Data Points #2 Incidence of diabetic foot ulcer and lower extremity amputation among Medicare beneficiaries, 2006 to 2008. In: Data Points Publication Series. Rockville (MD): Agency for Healthcare Research and Quality (US);2011. 22049565

[7] Jiang Y Wang X Xia L. A cohort study of diabetic patients and diabetic foot ulceration patients in China. Wound Repair Regen 2015;23:222–30. 25682850 10.1111/wrr.12263

[8] Lavery LA Armstrong DG Harkless LB. Classification of diabetic foot wounds. J Foot Ankle Surg 1996;35:528–31. 8986890 10.1016/s1067-2516(96)80125-6

[9] Li Z Xiaobing F. New indication for Extracorporeal Shock Wave Therapy (ESWT) approved by the FDA: treatment of diabetic foot ulcers. Infect Inflamm Repair 2017;18:204.

[10] Likan L Xiaogang L Jianjun H Fei G Jingchun Z. Mechanisms of extracorporeal shock wave therapy in promoting healing of diabetic footulcer. West China Med J 2020;35:363–66.

[11] Jeppesen SM Yderstraede KB Rasmussen BS Hanna M Lund L. Extracorporeal shockwave therapy in the treatment of chronic diabetic foot ulcers: a prospective randomised trial. J Wound Care 2016;25:641–49. 27827284 10.12968/jowc.2016.25.11.641

[12] Omar MT Alghadir A Al-Wahhabi KK Al-Askar AB. Efficacy of shock wave therapy on chronic diabetic foot ulcer: a single-blinded randomized controlled clinical trial. Diabetes Res Clin Pract 2014;106:548–54. 25451894 10.1016/j.diabres.2014.09.024

[13] Qiantong Q Yulong X Donghong J Changchun Y Xuebin W. Clinical observation on the efficacy of radial extracorporeal shock wave therapy in the treatment of diabetic foot. Mod Pract Med 2020;32:1136–37.

[14] Wu F Qi Z Pan B Tao R. Extracorporeal shock wave therapy (ESWT) favors healing of diabetic foot ulcers: a systematic review and meta-analysis. Diabetes Res Clin Pract 2024;217:111843. 39237040 10.1016/j.diabres.2024.111843

[15] Lipsky BA Senneville É Abbas ZG. Guidelines on the diagnosis and treatment of foot infection in persons with diabetes (IWGDF 2019 update). Diabetes/metab Res Rev 2020;36:e3280. 32176444 10.1002/dmrr.3280

[16] Mingliang L, and Gende G. An experimental study of the effects of extracorporeal shock wave treatment on osteonecrosis of the femoral head in adult rabbits. Chin J Phys Med Rehabil 2004;26:648–51.

[17] Vieweg J Weber HM Miller K Hautmann R. Female fertility following extracorporeal shock wave lithotripsy of distal ureteral calculi. J Urol 1992;148:1007–10. 1507317 10.1016/s0022-5347(17)36801-5

[18] Sistermann R Katthagen BD. Complications, side-effects and contraindications in the use of medium and high-energy extracorporeal shock waves in orthopedics. Zeitschrift Fur Orthopadie Und Ihre Grenzgebiete 1998;136:175–81. 9615982 10.1055/s-2008-1051302

[19] Haojun L Haiguang J Junyu Z. Chinese guidelines for extracorporeal shock wave therapy for bone and muscle diseases(2023 edition). China Med Front (Electronic Edition) 2023;15:1–20.

[20] Haiyan W Wenli L Jing L Jinzhou F. Effect of coordinated nursing intervention on mood disorders and ulcer healing in patients with diabetic foot ulcer complicated by depression. Chongqing Med 2017;46:4602–04.

[21] Jiaxin L Xicheng Z Qihang G Li W Guanqiang L. Research progress of chitosan and its complexes in the treatment of chronic lower limb ulcers. Chinese J of General Sur 2020;29:752–58.

[22] Abdissa D Adugna T Gerema U Dereje D. Prevalence of diabetic foot ulcer and associated factors among adult diabetic patients on follow-up clinic at Jimma medical center, Southwest Ethiopia, 2019: an institutional-based cross-sectional study. J Diabetes Res 2020;2020:4106383. 32258165 10.1155/2020/4106383 PMC7102459

[23] Wendao L Shiwei M Fanzhe M Xiaoqin F Hui X Gang C. Effects of endovascular angioplasty combined with oral traditional chinese medicine and foot bath on clinical symptoms and quality of life in elderly patients with diabetic foot. Chin J Gerontol 2014;34:6281–83.

[24] Cellular CAo. Foot ITTfD, Medicine TCoI, Branch BoCIP, Radiation NCfCMRo, treatment. clinical guidelines for comprehensive interventional management of the diabetic foot(9th Edition). J Interv Radiol 2024;33:341–54.

[25] Yimeng B Shanshan S Yangzhi W Zijian X Peng C. Regulatory networks of skin wound healing:regeneration,fibrosis,and chronic wounds. J China Med Univ 2024;53:1124–28.

[26] Dellon AL. The Dellon approach to neurolysis in the neuropathy patient with chronic nerve compression. Handchirurgie, Mikrochirurgie, plastische Chirurgie: organ der Deutschsprachigen Arbeitsgemeinschaft fur Handchirurgie. Organ der Deutschsprachigen Arbeitsgemeinschaft Fur Mikrochirurgie der Peripheren Nerven Und Gefasse 2008;40:351–60. 10.1055/s-2008-1039216 19051159

[27] Li L Rehemutula A Yu S Hongyu Z. Bone transfer combined with Dellon triple nerve decompression surgery for the treatment of diabetic foot. Chin J Orthop 2020;28:660–62.

[28] Tieying Q Jin H Qiuhong Z Jing Y Jin H. Influence of cleanout fluid of different temperature on epiphytic bacteria cleaning rate and healing rate of diabetic foot ulcer wound. Chin Gen Pract 2016;19:2169–73.

[29] Auersperg V Trieb K. Extracorporeal shock wave therapy: an update. EFORT Open Rev 2020;5:584–92. 33204500 10.1302/2058-5241.5.190067 PMC7608508

[30] Dizon JN Gonzalez-Suarez C Zamora MT Gambito ED. Effectiveness of extracorporeal shock wave therapy in chronic plantar fasciitis: a meta-analysis. Am J Phys Med Rehabil 2013;92:606–20. 23552334 10.1097/PHM.0b013e31828cd42b

[31] Simplicio CL Purita J Murrell W Santos GS Dos Santos RG Lana J. Extracorporeal shock wave therapy mechanisms in musculoskeletal regenerative medicine. J Clinl Orthopaedics Trauma 2020;11:S309–s18. 10.1016/j.jcot.2020.02.004 PMC7275282 32523286

[32] Yuan G Zhihan G Rui L Yonggang X Qinggang M Wei L. Research progress on extracorporeal shock wave therapy for chronic superficial wounds. Chin J Phys Med Rehabil 2019;41:545–49.

[33] Mazin Y Lemos C Paiva C Amaral Oliveira L Borges A Lopes T. The role of extracorporeal shock wave therapy in the treatment of muscle injuries: a systematic review. Cureus 2023;15:e44196. 37767244 10.7759/cureus.44196 PMC10521343

[34] Surace SJ Deitch J Johnston RV Buchbinder R. Shock wave therapy for rotator cuff disease with or without calcification. Cochrane Database Syst Rev 2020;3:Cd008962. 32128761 10.1002/14651858.CD008962.pub2 PMC7059880

[35] Gatz M Schweda S Betsch M. Line- and point-focused extracorporeal shock wave therapy for achilles tendinopathy: a placebo-controlled RCT study. Sports Health 2021;13:511–18. 33586526 10.1177/1941738121991791 PMC8404720

[36] Chen T Zhang P Chen J Hua Y Chen S. Long-term outcomes of anterior cruciate ligament reconstruction using either synthetics with remnant preservation or hamstring autografts: a 10-year longitudinal study. Am J Sports Med 2017;45:2739–50. 28892648 10.1177/0363546517721692

[37] Stania M Juras G Chmielewska D Polak A Kucio C Król P. Extracorporeal shock wave therapy for achilles tendinopathy. Biomed Res Int 2019;2019:3086910. 31950037 10.1155/2019/3086910 PMC6948318

[38] Song Y Che X Wang Z. A randomized trial of treatment for anterior cruciate ligament reconstruction by radial extracorporeal shock wave therapy. BMC Musculoskelet Disord 2024;25:57. 38216944 10.1186/s12891-024-07177-8 PMC10787473

[39] Mittermayr R Antonic V Hartinger J. Extracorporeal shock wave therapy (ESWT) for wound healing: technology, mechanisms, and clinical efficacy. Wound Repair Regen 2012;20:456–65. 22642362 10.1111/j.1524-475X.2012.00796.x

[40] Sundaram S Sellamuthu K Nagavelu K. Stimulation of angiogenesis using single-pulse low-pressure shock wave treatment. J Mol Med (Berlin, Germany) 2018;96:1177–87. 10.1007/s00109-018-1690-1 30155768

[41] Aglialoro F Abay A Yagci N. Mechanical stress induces Ca(2+)-Dependent signal transduction in erythroblasts and modulates erythropoiesis. Int J Mol Sci 2021;22:955. 33478008 10.3390/ijms22020955 PMC7835781

[42] Hitchman LH Totty JP Cai P Smith GE Carradice D Chetter IC. Extracorporeal shockwave therapy for diabetic foot ulcers: a feasibility study. J Wound Care 2023;32:182–92. 36930191 10.12968/jowc.2023.32.3.182

[43] Lim JZ Ng NS Thomas C. Prevention and treatment of diabetic foot ulcers. J R Soc Med 2017;110:104–09. 28116957 10.1177/0141076816688346 PMC5349377

[44] Semerci ÇV Çetinkaya ÖS. Patients with diabetic foot ulcers: a qualitative study of patient knowledge, experience, and encountered obstacles. J Tissue Viability 2024;33:571–78. 39068085 10.1016/j.jtv.2024.07.010

[45] Bus SA Sacco ICN Monteiro-Soares M. Guidelines on the prevention of foot ulcers in persons with diabetes (IWGDF 2023 update). Diabetes/metab Res Rev 2024;40:e3651. 37302121 10.1002/dmrr.3651

[46] Ruoqiong S. Research progress on wound debridement methods for diabetic foot ulcers. Nursing Research 2018;32:1833–35.

[47] Dayya D O’Neill OJ Huedo-Medina TB Habib N Moore J Iyer K. Debridement of diabetic foot ulcers. Adv Wound Care 2022;11:666–86. 10.1089/wound.2021.0016 PMC9527061 34376065

[48] Jianming J Genyuan Z Kaiwu L, and Chengshan L. Clinical application of extracorporeal shock wave therapy in degenerative bone and joint diseases in middle-aged and elderly patients. Chin J Phys Med Rehabil 2004;26:362–64.

[49] Chuanmin C He Z Lei W Xingang C. Advances in the diagnosis and treatment of urological system stones in 2019. Shanghai Med J 2020;43:341–46.

[50] Wei W Le Y Beijie Z. Clinical efficacy and safety of extracorporeal radial shockwave therapy for knee osteoarthritis. Shanghai Med J 2014;37:669–72.

[51] Dong A Yang L, and Tongjiang Y. Application of extracorporeal shock wave therapy in burn wound repair and post-burn scar treatment. Chin J Tissue Eng Res 2022;26:3265–72.

[52] Elias PM. Primary role of barrier dysfunction in the pathogenesis of atopic dermatitis. Exp Dermatol 2018;27:847–51. 29799646 10.1111/exd.13693

[53] Shi L Li Z Wang P Fan M Sun W. Irritant contact dermatitis following extracorporeal shockwave therapy: a case report. Ann Palliat Med 2021;10:7083–87. 33548985 10.21037/apm-20-1830

[54] GuangHua L Xiaobin F Yanfei L. Laboratory examination and clinical analysis of adult acquired vitamin K deficiency. J Southern Med Univ 2009;29:1716–17.

[55] Lianwei S Wei W. Radial extracorporeal shockwave combined with resistance exercise for treating senile primary sarcopenia. Chin J Tissue Eng Res 2023;27:1647–52.

[56] Run Z Quanbing Z Yun Z. Analysis of therapeutic effect of extracorporeal shock wave with different intensity on traumatic extended knee joint contracture. Chin J Rehabil Med 2024;39:1625–31.

[57] Jingwen K Wei S Changshui W Meirong L Yun L Li Z. Effects of shock wave on proliferation and migration of HaCat cells in chronic diabeticwound cell model. J PLA Med Acad 2022;43:1253–8+305.

[58] Moretti B Notarnicola A Maggio G. The management of neuropathic ulcers of the foot in diabetes by shock wave therapy. BMC Musculoskelet Disord 2009;10:54. 19473538 10.1186/1471-2474-10-54 PMC2693140

[59] Schroeder AN Tenforde AS Jelsing EJ. Extracorporeal shockwave therapy in the management of sports medicine injuries. Curr Sports Med Rep 2021;20:298–305. 34099607 10.1249/JSR.0000000000000851

[60] Yahata K Kanno H Ozawa H. Low-energy extracorporeal shock wave therapy for promotion of vascular endothelial growth factor expression and angiogenesis and improvement of locomotor and sensory functions after spinal cord injury. J Neurosurg Spine 2016;25:745–55. 27367940 10.3171/2016.4.SPINE15923

[61] Zhang J Kang N Yu X Ma Y Pang X. Radial extracorporeal shock wave therapy enhances the proliferation and differentiation of neural stem cells by Notch, PI3K/AKT, and Wnt/β-catenin signaling. Sci Rep 2017;7:15321. 29127399 10.1038/s41598-017-15662-5 PMC5681501

[62] Feng L Lifan Z Weidong W Jiagang W. Efficacy of extracorporeal shock wave therapy combined with spinal manipulation for treating lumbar disc herniation in the elderly. Chinese J Geriatrics 2024;44:2646–49.

[63] Hongbai L Xiaodong H Liming Q Mingsheng Z Zhenqiang H Fan C. Efficacy of extracorporeal shock wave therapy for patellofemoral chondromalacia. Guangdong Med J 2013;34:244–46.

[64] Bizhi L Yumeng W Daan Z. Exploration of the mechanism of extracorporeal shock wave therapy in treating chronic skeletal muscle injury in rats based on the PI3K-AKT signaling pathway. Chin J Gerontol 2023;43:2475–80.

[65] Wang CJ Kuo YR Wu RW. Extracorporeal shockwave treatment for chronic diabetic foot ulcers. J Surg Res 2009;152:96–103. 18619622 10.1016/j.jss.2008.01.026

[66] Porso M Loreti S Nusca SM. Defocused shock wave therapy for chronic soft tissue wounds in the lower limbs: a pilot study. Ultrasound Med Biol 2017;43:362–69. 27745716 10.1016/j.ultrasmedbio.2016.08.038

[67] Yunxiang D Jia Y Jiayou Z. Analysis of short-term efficacy of extracorporeal shock wave therapy for knee osteoarthritis. Chin J Rehabil Med 2016;31:917–19.

[68] Yunshen G Shiyi C Yunxia L. Report on 35 cases of extracorporeal shock wave therapy for soft tissue injuries of the shoulder tendon. Chin J Sports Med 2011;30:1026–29.

[69] Kamelger F Oehlbauer M Piza-Katzer H Meirer R. Extracorporeal shock wave treatment in ischemic tissues: what is the appropriate number of shock wave impulses? J Reconstr Microsurg 2010;26:117–21. 20013593 10.1055/s-0029-1243296

[70] Larking AM Duport S Clinton M Hardy M Andrews K. Randomized control of extracorporeal shock wave therapy versus placebo for chronic decubitus ulceration. Clin Rehabilitat 2010;24:222–29. 10.1177/0269215509346083 20156981

[71] Huidong L Weiling S Shuhui L. Application of growth factors combined with moist dressings in chronic wound care. Nursing Research 2019;33:4144–45.

[72] Lan Z Kar R Chwatko M Shoga E Cosgriff-Hernandez E. High porosity PEG-based hydrogel foams with self-tuning moisture balance as chronic wound dressings. J Biomed Mater Res Part A 2023;111:465–77. 10.1002/jbm.a.37498 PMC11542385 36606332

[73] Yi Z Chao C Pingping W. Pathological mechanisms of delayed wound healing in diabetic foot ulcers. J Clin Dermatol 2025;54:48–51.

[74] Xiulin H Ketao W Xiaoying Z. Prognostic analysis of pneumatic ballistic dispersive shock wave and ultrasound-guided corticosteroid injection in the treatment of plantar fasciitis. J Southern Med Univ 2018;38:135–40. 10.3969/j.issn.1673-4254.2018.02.03 PMC6743879 29502050

[75] Xingyi L Jiren W Hongwei W Lei H. Efficacy of extracorporeal shock wave therapy for elderly patients with heel pain syndrome. Chinese J Geriatrics 2013;33:2432–33.

[76] Wang CJ Wu RW Yang YJ. Treatment of diabetic foot ulcers: a comparative study of extracorporeal shockwave therapy and hyperbaric oxygen therapy. Diabetes Res Clin Pract 2011;92:187–93. 21310502 10.1016/j.diabres.2011.01.019

[77] Jianying Z Xihu Y Jingjia Y. Efficacy observation and preliminary mechanism study of extracorporeal shock wave therapy for diabetic foot. Chin Med Device Inf 2020;26:43–4+161.

[78] Chen P Vilorio NC Dhatariya K. Guidelines on interventions to enhance healing of foot ulcers in people with diabetes(IWGDF 2023 update):Part of the 2023 IWGDF guidelines on the prevention and management of diabetes-related foot disease. Infect Inflamm Repair 2024;25:23–51. 10.1002/dmrr.3644 37232034

[79] Xiaoyan Z Lizhu G Youcai Z. The effect of basic treatment combined with red-blue light and Wuhuang solution on wound healing and inflammatory factors in diabetic foot patients. Diabetes New World 2024;27:173–5+9.

[80] Ying X Zhen H Zirui L Chao L. Clinical efficacy observation of extracorporeal shock wave therapy combined with WIRA in the treatment of radial styloid tenosynovitis. Chin J Rehabil Med 2019;34:1092–94.

[81] Blume PA Walters J Payne W Ayala J Lantis J. Comparison of negative pressure wound therapy using vacuum-assisted closure with advanced moist wound therapy in the treatment of diabetic foot ulcers: a multicenter randomized controlled trial. Diabetes Care 2008;31:631–36. 18162494 10.2337/dc07-2196

[82] Gethin G Cowman S Kolbach DN. Debridement for venous leg ulcers. Cochrane Database Syst Rev 2015;2015:Cd008599. 26368002 10.1002/14651858.CD008599.pub2 PMC6486053

[83] Ji S Liu X Huang J. Consensus on the application of negative pressure wound therapy of diabetic foot wounds. Burns Trauma 2021;9:tkab018. 34212064 10.1093/burnst/tkab018 PMC8240517

[84] O’Meara S Al-Kurdi D Ologun Y Ovington LG Martyn-St James M Richardson R. Antibiotics and antiseptics for venous leg ulcers. Cochrane Database Syst Rev 2014;2014:Cd003557. 24408354 10.1002/14651858.CD003557.pub5 PMC10580125

[85] Wang CJ Cheng JH Kuo YR Schaden W Mittermayr R. Extracorporeal shockwave therapy in diabetic foot ulcers. Int J Surg (London, England) 2015;24:207–09. 10.1016/j.ijsu.2015.06.024 26079500

[86] Wei S Li Z Meirong L Xiaobing F. A review on the mechanisms of extracorporeal shock wave therapy in promoting tissue repair and regeneration. J PLA Med Acad 2020;41:638–43.

